# Laparoscopic-Assisted Transgastric Endoscopic Retrograde Cholangiography (LTG-ERCP): An Option for Managing Choledocholithiasis in a Patient With Prior Bariatric Surgery at a Community Hospital

**DOI:** 10.7759/cureus.92112

**Published:** 2025-09-12

**Authors:** Seemab Fatima, Maham Tariq, Muhammad Hammad Ashraf, Hafsa Riaz, Muhammad Tayyeb, Shazia M Shah

**Affiliations:** 1 Internal Medicine, Services Hospital Lahore, Lahore, PAK; 2 Internal Medicine, BronxCare Health System, New York City, USA; 3 Hospital Medicine, St. Francis Hospital, Wilmington, USA; 4 Internal Medicine, Rutgers Health/Monmouth Medical Center, Long Branch, USA

**Keywords:** bariatric surgery, choledocholithiasis, community teaching hospital, double balloon endoscopy, endoscopic retrograde cholangiography, laparoscopic roux-en-y gastric bypass

## Abstract

Endoscopic retrograde cholangiography (ERCP) has become a mainstay in managing choledocholithiasis. Conventional endoscopic access to the ampulla of Vater becomes impossible after Roux-en-Y gastric bypass (RYGB) due to modified anatomy. In specialized centers with advanced gastrointestinal capabilities, techniques like double-balloon endoscopy can be utilized for biliary decompression. However, facilitating patient transfer to these centers can be difficult due to a common postoperative issue in a large population. Laparoscopic transgastric (LTG)-ERCP has been gaining popularity because of its high success rates and ability to carry it out in community healthcare settings. Here we present a case of choledocholithiasis in an RYGB patient successfully managed with LTG-ERCP at a community hospital. Our patient was a 51-year-old female with a history of sleeve gastrectomy with conversion to gastric bypass who presented to the emergency department for evaluation of severe epigastric pain and nausea. Laboratory testing was consistent with elevated bilirubin and alkaline phosphatase levels (1.5 mg/dl and 140 U/L, respectively). Abdominal ultrasound showed biliary sludge in the common bile duct (CBD). Subsequent magnetic resonance cholangiopancreatography (MRCP) showed dilatation of the CBD measuring up to 1 cm with multiple stones in the biliary system. After a multidisciplinary discussion between the GI and surgery teams, an LTG-ERCP along with cholecystectomy was performed, and CBD stones were successfully removed, resulting in the resolution of the patient’s symptoms, and the patient was discharged home without any complications. LTG-ERCP is considered a safe and effective approach for managing biliary obstruction after RYGB. This procedure has the advantage of endoscopic biliary decompression and cholecystectomy being performed in a single setting.

## Introduction

Biliary lithiasis and related complications are common after bariatric surgery due to rapid weight loss, particularly after Roux-en-Y bypass surgery (RYGB) [1]. Endoscopic retrograde cholangiography (ERCP) has become a mainstay in the management of choledocholithiasis, as it offers both diagnostic and therapeutic capabilities for biliary decompression. However, conventional endoscopic access to the ampulla of Vater is not feasible after RYGB due to surgically altered anatomy, as RYGB separates the gastric pouch from the duodenum, excludes the native stomach and duodenum from the alimentary tract, and creates a long, tortuous route to the ampulla. Double-balloon enteroscopy-assisted ERCP (EA-ERCP) is therefore utilized in a few tertiary care centers with advanced gastrointestinal capabilities for biliary decompression [2]. Although this procedure is efficacious, facilitating patient transfers can be costly and time-consuming. Several alternative techniques have emerged to address this need in routine clinical settings, which include percutaneous transhepatic cholangiography, endoscopic ultrasound-directed transgastric ERCP (EDGE), and laparoscopic transgastric (LTG)-ERCP [3]. LTG-ERCP is performed in certain patient populations, including those with RYGB-altered anatomy, biliary obstruction with chemicals, imaging evidence of common bile duct (CBD) stones, and patients with the ability to tolerate general anesthesia [4]. LTG-ERCP has gained popularity due to its high success rates and feasibility in community healthcare settings equipped with basic surgical and endoscopic infrastructure. This technique involves laparoscopic access to the excluded gastric remnant through a surgical gastrostomy, allowing an endoscope to be introduced directly into the remnant stomach and guided to the biliary tree under direct visualization [5]. We hereby present a case of choledocholithiasis in an RYGB patient successfully managed with LTG-ERCP at a community hospital.

## Case presentation

**Figure 1:**
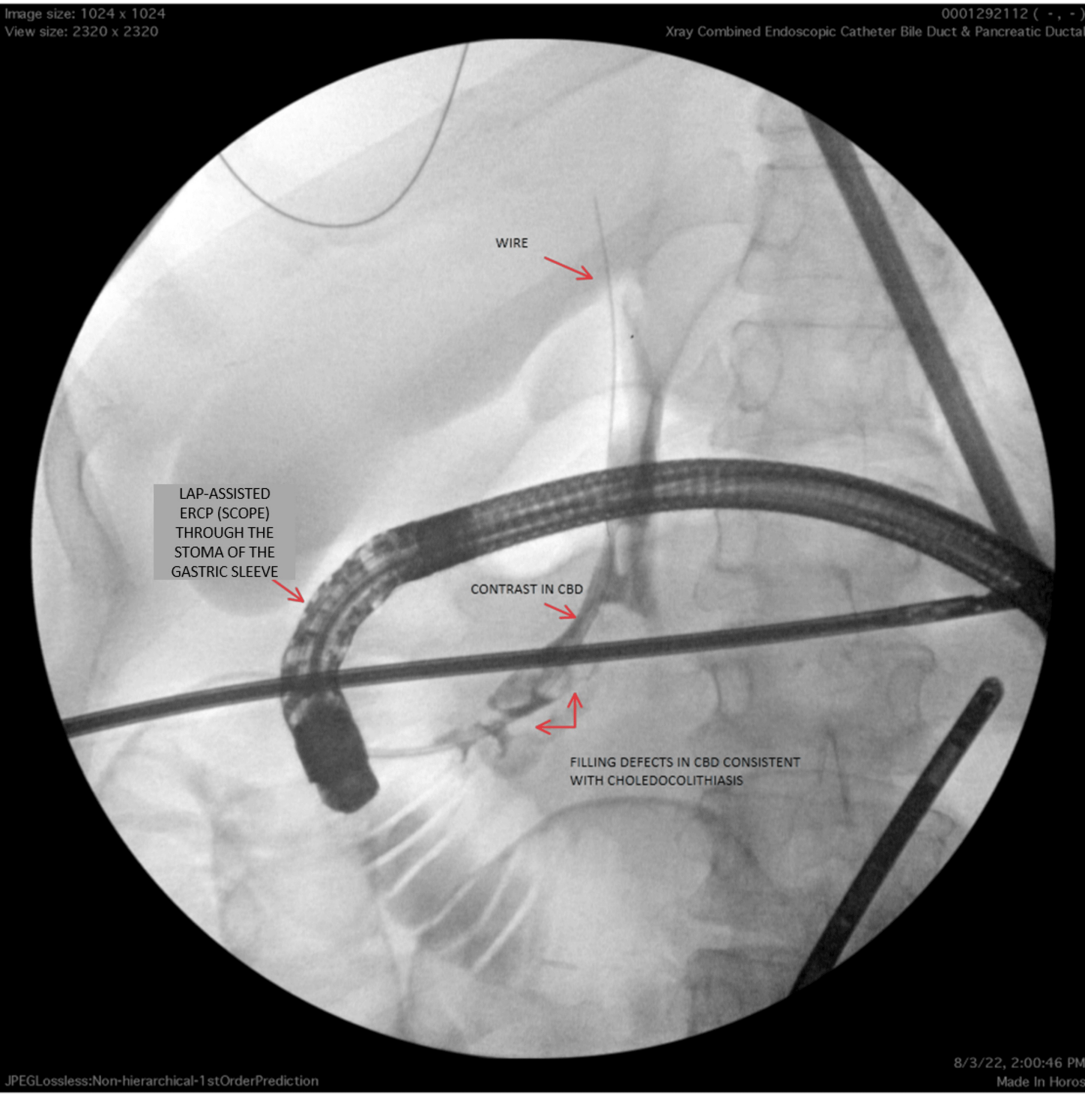
Pre-procedure image: filling defect seen in the CBD consistent with choledocholithiasis Lap: laparoscopy; ERCP: endoscopic retrograde cholangiography; CBD: common bile duct

**Figure 2:**
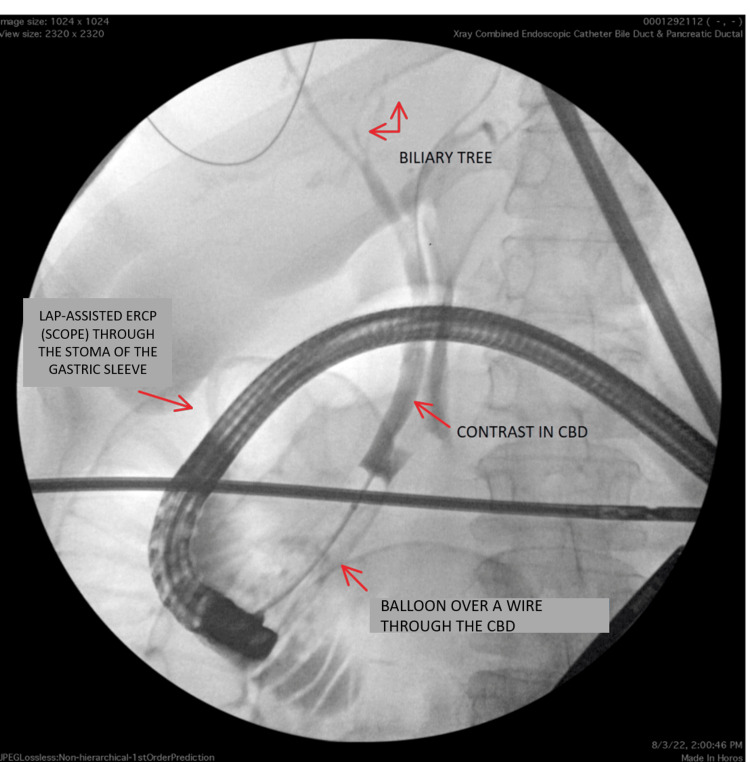
Post LTG-ERCP image: contrast can be seen in the CBD with no evidence of filling defects Lap: laparoscopy; LTG: laparoscopic transgastric; ERCP: endoscopic retrograde cholangiography; CBD: common bile duct

Our patient was a 51-year-old female with a medical history significant for sleeve gastrectomy in 2016 with conversion to gastric bypass in 2021 due to persistent heartburn, who presented to the emergency department for the evaluation of severe epigastric pain and nausea. On arrival to the ED, the patient’s blood pressure was 123/59 mmHg, heart rate was 62 beats per minute (bpm), and temperature was 98.4°F, saturating 96% on room air. Physical examination was significant for scleral icterus and right upper quadrant abdominal tenderness. Laboratory studies were significant for mildly elevated bilirubin and alkaline phosphatase levels (Table 1). Ultrasound of the abdomen showed biliary sludge in the CBD. Subsequent magnetic resonance cholangiopancreatography (MRCP) showed dilatation of the CBD measuring up to 1 cm with multiple stones in the biliary system, along with dilatation of the intrahepatic biliary system (Figure 1). After a multidisciplinary discussion between the gastroenterology and surgery teams, an LTG-ERCP, along with cholecystectomy in a single setting, was planned. The general surgery team created laparoscopic access to the excluded stomach, through which a trocar was placed, which allowed the gastroenterologist to insert a conventional side-viewing duodenoscope directly into the remnant stomach and advance into the duodenum for further intervention. For this procedure, the Olympus TJF-Q180V therapeutic duodenoscope (Olympus Corporation, Center Valley, PA) was used. used. Intraoperatively, CBD stones were found on cholangiography and removed successfully during the procedure (Figure 2). The procedure resulted in the resolution of the patient’s symptoms, and the patient was discharged on the second day without any complications.

**Table 1:** The patient's liver function test results

Test	Value	Reference Range
Total Bilirubin	2.7 mg/dL	0.1 - 1.2 mg/dL
Direct Bilirubin	1.5 mg/dL	0.0 - 0.3 mg/dL
Aspartate Aminotransferase (Serum Glutamic-Oxaloacetic Transaminase)	90 U/L	10 - 40 U/L
Alanine Aminotransferase (Serum Glutamic Pyruvate Transaminase)	87 U/L	7 - 56 U/L
Alkaline Phosphatase	140 U/L	44 - 147 U/L

## Discussion

**Figure 3:**
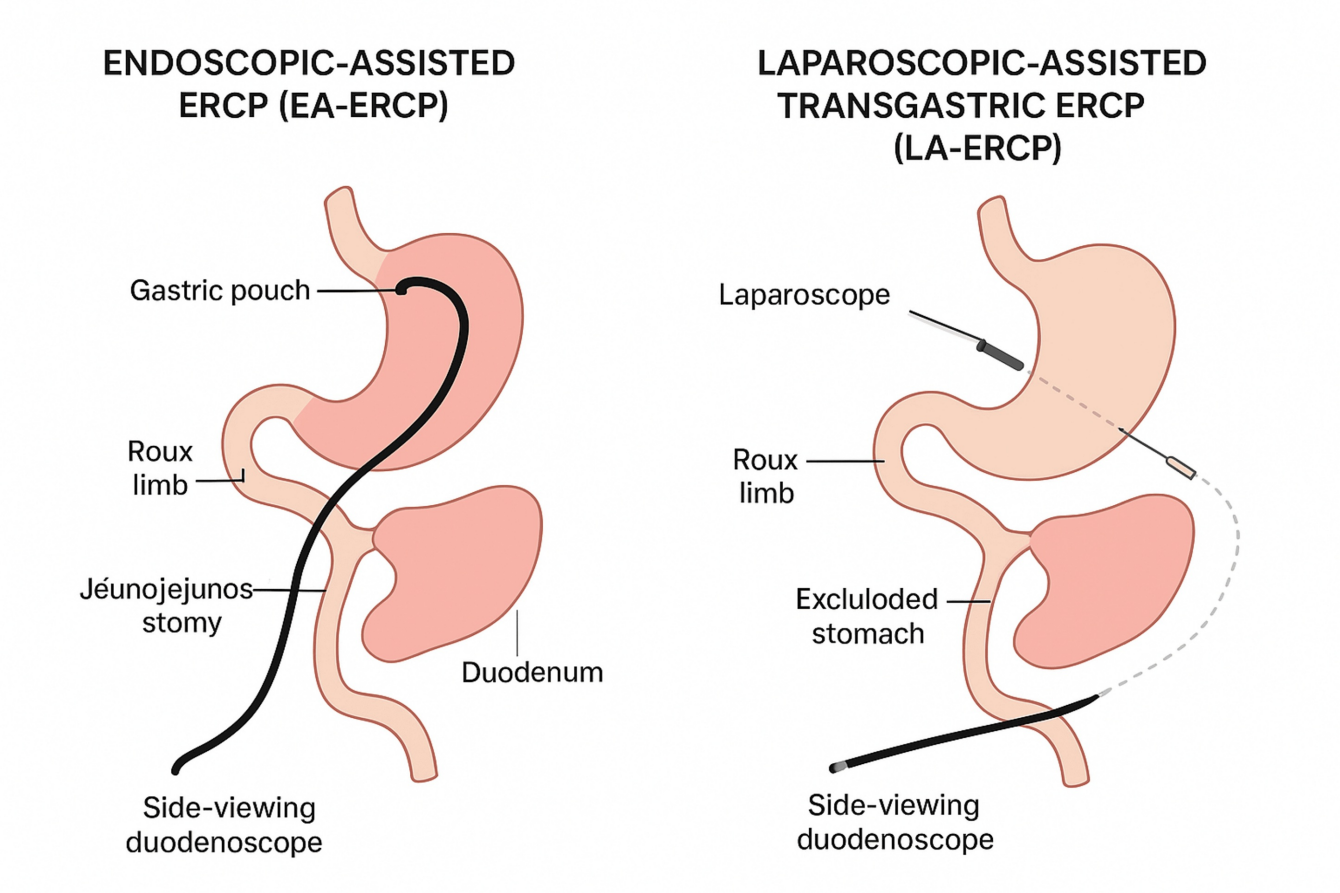
Difference between the techniques of the two commonly performed procedures in in RYGB patients. ERCP: endoscopic retrograde cholangiography; RYGB: Roux-en-Y gastric bypass This figure has been created by the authors.

Gastric bypass surgery can lead to rapid weight loss, which increases the risk of developing issues related to the gallbladder. This was reflected in a recent monocentric prospective study by Coupaye et al. that deduced that losing more than 30 kilograms of weight over six months after RYBG was associated with the development of gallstones [6]. Similar results are reported by Weinsier et al.; losing weight at a rate greater than 1.5 kilograms a week was associated with the development of gallstones [7]. Patients developing biliary complications, such as choledocholithiasis and cholangitis, after gastric bypass surgery may require specialized techniques to access the biliary tract. Two of the most common approaches are laparoscopic-assisted ERCP (LA-ERCP), which involves creating a gastrostomy for endoscopy access, and EA-ERCP, which uses an overtube-based enteroscopy to access the biliary system [8]. EA-ERCP involves using an overtube-based (single balloon, double balloon, or spiral) enteroscopy in which the endoscope/overtube combination is passed through the mouth via the Roux limb to access the biliary system. After reaching enteroenterostomy, the pancreaticobiliary limb is accessed in retrograde fashion to reach the papilla. Then, ERCP is carried out via the forward-viewing, 200-cm-long enteroscope (therapeutic channel 2.8 mm) using dedicated “long” accessories (Figure 3) [9].

Laparoscope-assisted transgastric endoscopy was first described in 2002 but had not been truly proven effective until recent times, when it was utilized in clinical practice and proved to be beneficial for patients who had undergone RYGB for severe obesity. This technique has several variations, including the number of access ports used, ranging from as few as two to an optimal choice of three, depending on the case and the surgeon's preference. The three-port access is considered optimal for LA-ERCP because it provides adequate working space and instrument triangulation for safe manipulation and leads to quicker recovery. The placement of these ports is adaptable and considers the patient's unique anatomy and prior surgeries. The location of the gastrotomy is determined by placing three or four sutures in a triangular or diamond-shaped configuration, ensuring adequate retraction and exposure during the procedure. Additionally, there are options for how the gastrotomy is closed after the procedure. The preferred method is a double-layered closure, though in special circumstances, a single-layer or stapled closure may be employed. Furthermore, in certain situations, a gastrostomy tube may be retained to support enteral feeding or potential future access to the gastric remnant. These variations highlight the adaptability and customization available in laparoscopic-assisted trans-gastric endoscopy to cater to the specific requirements of individual patients [10]. Mauricio Gonzalez-Urquijo et al. have introduced a technique that involves creating a single purse-string suture 2 cm above the gastric incision to aid in endoscope insertion, thus providing the necessary traction. This technique differs from that described by Fachiano et al., who lift the stomach and suture it to the abdominal wall while inserting the endoscope through a trocar within the stomach. This maneuver is thought to prevent gastric content leakage into the abdominal cavity; however, the current scientific data to support its use is insufficient. In contrast, the approach discussed in this context involves passing the endoscope directly through the gastrostomy without requiring an intragastric trocar. This method avoids the need to prolong the procedure's duration and has not been associated with postoperative complications. Various approaches to this procedure have been published between 2010 and the present day, highlighting the ongoing development and diversity in the field [11]. A meta-analysis by Ayoub et al. found that transgastric LA-ERCP had a much higher success rate (therapeutic success 97.9%; 95% CI: 96.7-98.7%) compared to EA-ERCP, which had a lower success rate (therapeutic success 73.2%; 95% CI: 62.5-82.6%). However, this higher success in LA-ERCP is associated with more adverse events (AEs) and a longer procedure time [12]. Saad et al. found that the overall technical success rate for LA-ERCP was quite high and comparable to the success rate of ERCP in individuals with normal gastrointestinal anatomy. This indicates that LA-ERCP is effective at successfully navigating to the intended duct, thus leading to a high rate of successful clinical outcomes. It's essential to consider these LA-ERCP results in comparison to other techniques used in managing patients who've had gastric bypass surgery, as it can impact the choice of the procedure and the healthcare provided to these patients [13]. da Ponte-Neto et al. suggested the reason for the high success rates in LA-ERCP: firstly, LA-ERCP benefits from using standard duodenoscopes, which provide better angled views of the papilla, and utilizes appropriate ERCP tools. Secondly, it employs the use of an elevator, hence providing better access to the papilla. These advantages are deficient in balloon enteroscopy-based techniques [14].

James et al. compared the cost-effectiveness of different procedures (EA-ERCP, EDGE, and LA-ERCP) for treating pancreaticobiliary issues in patients who've had gastric bypass surgery in the USA. They based their study on previously published retrospective studies rather than conducting new ones. They deduced that EDGE was the most cost-effective option for managing these conditions in post-gastric bypass patients when compared to device-assisted enteroscopy (DAE)-ERCP and LA-ERCP. EDGE resulted in the lowest total costs and highest total quality-adjusted life-years (QALY) for a total of $5188/QALY in comparison with EA-ERCP and LA-ERCP, which cost $11,263 and $34,259, respectively. They also confirmed the robustness of this conclusion through sensitivity analysis, showing that it held up even when important model parameters were changed [15]. Another study by Wang et al. evaluated the cost associated with the procedure and hospital stay, highlighting the cost advantages of EDGE over LA-ERCP, though it had similar success rates [16].

A meta-analysis conducted by Ayoub et al. found that LA-ERCP tends to have a higher rate of AEs. These AEs are primarily due to the laparoscopic part of the procedure, particularly infections and bleeding, rather than the ERCP itself. Interestingly, the combined rates of ERCP-related AEs were similar between both LA-ERCP and EA-ERCP. Many of the AEs related to laparoscopy were relatively minor and resolved on their own; however, some were more severe, including cases requiring transfusions due to bleeding, the formation of abscesses in the abdominal cavity, and tension pneumothorax. This emphasizes the need for a personalized approach, considering the patient's medical conditions and unique characteristics when selecting the most suitable ERCP method. As expected, EA-ERCP had a shorter average procedure time compared to LA-ERCP (100.5 minutes vs. 158.4 minutes), primarily because LA-ERCP requires additional time for laparoscopic access to the stomach [12]. While the time savings with EA-ERCP might seem appealing, especially for busy endoscopy units, it's essential to weigh this against the potential for lower overall success rates of EA-ERCP compared to LA-ERCP. Moreover, if an attempt at EA-ERCP fails, it may necessitate additional procedures like LA-ERCP or percutaneous transhepatic biliary drainage. Each of these has its associated costs, time commitments, and the potential for AEs. It's crucial to be cautious when interpreting the aggregated results regarding procedural time, as only four out of 12 EA-ERCP studies reported these data, and these studies had varying types of enteroscopy and operator experience.

## Conclusions

LTG-ERCP seems to be an effective solution for managing choledocholithiasis in post-RYGB patients, especially in community hospital settings where advanced endoscopic techniques are not available. Our case focuses on the feasibility, safety, and success of LTG-ERCP when performed through multidisciplinary collaboration. Though LTG-ERCP carries higher procedural complexity and adverse effects when compared to the endoscopic procedures, its success rate and feasibility for community hospital infrastructure make it a valuable option. If we implement this technique in the community hospitals nationwide, it could significantly improve the outcomes and access to care for the increasing bariatric patient population. 
